# The genealogical method in epistemology

**DOI:** 10.1007/s11229-018-1675-1

**Published:** 2018-01-16

**Authors:** Martin Kusch, Robin McKenna

**Affiliations:** University of Vienna, Universitätsstrasse 7 (NIG), 1010 Wien, Austria

**Keywords:** Edward Craig, Genealogy, Philosophical methodology, Knowledge, Relativism

## Abstract

In 1990 Edward Craig published a book called *Knowledge and the State of Nature *in which he introduced and defended a genealogical approach to epistemology. In recent years Craig’s book has attracted a lot of attention, and his distinctive approach has been put to a wide range of uses including anti-realist metaepistemology, contextualism, relativism, anti-luck virtue epistemology, epistemic injustice, value of knowledge, pragmatism and virtue epistemology. While the number of objections to Craig’s approach has accumulated, there has been no sustained attempt to develop answers to these objections. In this paper we provide answers to seven important objections in the literature.

## Introduction

This paper is a contribution to current debates over philosophical methodology in the analytic tradition. We shall discuss a method first suggested and developed in Edward Craig’s *Knowledge and the State of Nature* (1990), and labelled, by different interpreters, ‘state-of-nature epistemology’, ‘conceptual synthesis’, ‘function-first epistemology’, ‘practical explication’, or ‘genealogy’. We prefer the last term both because it is the one on which Craig eventually settled, and because it hints at continuities across the ‘Analytic/Continental’ divide: Craig himself mentions Nietzsche, Foucault, Hobbes, and Hume as amongst his sources (Craig 2007). In addition to Craig’s 1990-book, we shall also draw on his German-language volume *Was wir wissen können* (1993), as well as his 2007 ‘retrospective’, ‘Genealogies and the State of Nature’.

Craig’s genealogy of the concept of *knowledge* starts by asking which fundamental and universal human needs are served by our social institution of knowledge attributions.^1^ His working hypothesis is that the key factor is our need to ‘flag good informants’. Craig seeks to vindicate this hypothesis by showing how it predicts—or better: ‘retrodicts’—both central features of our knowledge-talk and many existing analyses of our concept of *knowledge*. For purposes of exposition Craig couches his investigation in the garb of a historical thesis about how the concept *knowledge* has evolved.

Craig’s methodology has attracted a good number of followers and critics.^2^ And his work has been put to a number of different uses: for instance, in anti-realist metaepistemology (Dogramaci 2012; Neta 2006), contextualism (Hannon 2013; Henderson 2009; McKenna 2013), relativism (MacFarlane 2014), anti-luck virtue epistemology (Pritchard 2012), epistemic injustice (Fricker 2007), value of knowledge (Kusch 2009), pragmatism (Williams 2015), and virtue epistemology (Greco 2007). Rather than add a new project to these existing ones, in this paper we focus instead on the growing number of *objections* to Craig’s approach. Some of these objections concern fundamental aspects of the project, and, absent good answers, there is reason to suspect that the genealogical method itself is fundamentally flawed. We shall try to sketch what such ‘good answers’ might look like.

## Summary of the genealogical method

In his 1993 German-language book Craig situates his project in close proximity to two somewhat unlikely bedfellows: Wittgenstein and natural science (1993: p. 37). Wittgenstein is an ally since he opposes conceptual analysis in terms of necessary and sufficient conditions, studies the function of concepts, and introduces the category of family-resemblance concepts. Craig’s project has affinities with natural science in its method of hypothesis testing, the search for explanation, and a focus on evolution. Going beyond his wording, we would add *model-building* to the list: the building of simplified (and possibly even distorting) models of complex target systems.

Craig’s model-construction has two stages: the first focuses on the ‘epistemic state of nature’, that is, a small community of language-using humans, engaging primarily in face-to-face communication, who are co-operative, dependent upon one another for information, and of unequal skills and talents. The central question regarding this state of nature is: Why would a concept like *knowledge* be introduced under these idealised—simplified and distorted—conditions? Craig answers that people in this situation have a salient need, to wit, the need to pick out and ‘flag good informants’. And the concept used to flag good informants is the core—or one central aspect—of *knowledge*.

In the state of nature, individuals depend upon one another for information. Distinguish between the roles of ‘inquirer’ and ‘informant’. The inquirer needs information that she is currently unable to directly obtain herself; the informant offers such information. Inquirers must be able to separate good from bad informants. And it is natural to assume that meeting this need will involve concepts. Assume that the concept of a *protoknower* is the central conceptual tool for dealing with this problem. Which conceptual components should *protoknower* contain? What should we hypothesize our imaginary ancestors to want this concept for? Craig’s answers are that our ancestors want this concept as a tag for good informants and that the concept *protoknower* (*whetherp*) comprises the following elements:

(i) Being as likely to be right about *p* as the inquirer’s current needs require;
(ii) Being honest;
(iii) Being able to make the inquirer believe that *p*;
(iv) Being accessible to the inquirer here and now;
(v) Being understandable to the inquirer; and
(vi) Being detectable as a good informant concerning *p* by the inquirer.

To elaborate briefly on (vi), the inquirer needs to find ‘indicator-properties’ that she can detect and that correlate closely and in a law-like fashion with holding a true belief, or telling the truth, as to whether *p* (1990: pp. 25, 135). ‘Being at the top of a tree’ might be such a property for some inquirers in the state of nature when *p* is the proposition that a tiger is approaching the village. Usually more than one property will be involved. The properties that make Fred a medical protoknower are not one but many.

Craig is adamant that (i) to (vi) are not necessary and sufficient conditions. While all these elements are present in prototypical situations, the concept has a use even when some of the elements are missing. Finally, *protoknowledge* differs from *knowledge* in that: (a) only the former is closely tied to testimony; (b) *protoknowledge* is indexed to the capacities and needs of specific inquirers (1990: p. 90); (c) *protoknowledge* can be ascribed only to others but not to oneself; and (d) protoknowledge is not undermined by accident or luck: users of *protoknowledge *lack the intellectual sophistication to distinguish between accidental and non-accidental fulfillment of the conditions of *protoknowledge*.

Craig goes to great length to show that his model of the epistemic state of nature passes the test of (what the philosophy of scientific models calls) ‘external validation’. He does so by arguing that his model predicts and explains several features of our concept(s) of *knowledge* that have been identified in various philosophical theories. For instance,

- uses of ‘knowledge’ without belief (Radford) (Craig 1990: pp. 15–16)
- the role of counterfactuals (Nozick, Dretske) (Craig 1990: Ch. III)
- the role of causal relations (Goldman) (Craig 1990: Ch. IV)
- the role of methods (reliabilism) (Craig 1990: Ch. IV)
- the role of justifying reasons (internalism about justification) (Craig 1990: Ch. VIII)
- that all analyses have counterexamples (Gettier) (Craig 1990: Ch. VI) and
- the contextual variation in standards (Unger) (Craig 1990: Ch. XII).

These theories are often seen as excluding one another, but Craig thinks that his model can partially vindicate all of them: they contradict each other only if we over-generalise them.

This brings us to the second half of the genealogical just-so story: the hypothetical social-historical narrative that takes us from *protoknowledge* to *knowledge*. Craig speaks of this development as a process of ‘objectivization’ of *protoknowledge*. Key steps in this objectivization are the following. First, *protoknowledge* comes to be used in self-ascription. In response to the question ‘who knows whether *p*?’ group members start to investigate their own indicator-properties. Second, inquirers begin to recommend informants to others. This can be done in a helpful manner only if the perspectival or indexical character of *protoknowledge* is weakened. The recommended informant must be good in the eyes of both the recommender and the recipient of the recommendation. Further movement in this dimension—recommending an informant to ever more inquirers—makes protoknowledge increasingly harder to get. The endpoint is the idea of “someone who is a good informant as to whether *p* whatever the particular circumstances of the inquirer ... That means someone with a very high degree of reliability, someone who is very likely to be right—for he must be acceptable even to a very demanding inquirer” (1990: p. 91). And a very demanding inquirer will not accept epistemic luck or accident. Third, inquirers begin to use ‘being recommended’ as an indicator property. This move dilutes the original detectability requirement. Inquirers begin calling someone a ‘protoknower’ even when none of the original ‘natural’ indicator-properties is in sight. Fourth, in the context of group action inquirers cease to care whether the needed information is accessible to them as individuals; they are satisfied if it is accessible to someone in the group. As a result they will speak of ‘protoknowledge’ even outside the context of testimony. The process of objectivization ends up with our concept of *knowledge*: “The concept of knowing ... lies at the objectivised end of the process; we can explain why there is such an end, and why it should be found worth marking in language” (Craig 1990: pp. 90–91).

The second stage of Craig’s model construction adds a *dynamic* dimension to the state-of-nature. The dynamic model takes the epistemic state of nature as its starting point and tracks how the concept *knowledge *would evolve and diversify as the simplifications and distortions of the state of nature are removed step by step. This suggests that the dynamic model is really a form of ‘de-idealisation’.

The dynamic model too needs to pass muster as far as external validation is concerned. Craig suggests that it correctly predicts, or at least makes sense of,

- contexts with very high epistemic standards (1990: Ch. X),
- the distinction between know-how and know-that (1990: Ch. XVII),
- intuitions about lottery propositions (1990: Ch. XI), and
- our conflicting intuitions about epistemic scepticism (1990: Ch. XII–XIII).

As Craig emphasises more clearly in 2007 than in 1990 or 1993, this is not to be taken as a historical thesis: the epistemic state of nature is not a historical period ‘like the Pleistocene’. It is rather a ubiquitous and important type of social-epistemic situation that one is likely to find in all human communities, past and present. Or, as Craig himself puts the point:

... the circumstances that favor the formation of the concept of [proto-] knowledge still exist... otherwise I would have no support for my thesis that the method reveals the core of the concept as it is to be found now (2007: p. 191).

This suggests that what the two models present as different stages in the historical development of *knowledge* are really different types of situation that we experience from day-to-day. In some situations, we are still in the ‘state-of-nature’, in other situations we are at various stages of the process of objectivization. Note however that this interpretation of Craig shows that his talk of a ‘core’ of *knowledge* might be misleading: if the other uses co-exist, why assume that ‘flagging good informants’ is more fundamental than the other uses? Or put differently, why assume that the right model for the conceptual development is an avalanche rather than a phylogenetic tree? The avalanche model suggests a small conceptual ‘stone’ (i.e. *protoknowledge*) rolling down the snowy (semantic) mountain (of objectivization), in the process putting on layer after layer of further conceptual features (to reach *knowledge*). The phylogenetic tree is without a core. We do not think of *homo erectus* as the core or essence of *homo sapiens* just because *homo sapiens* developed out of *homo erectus*. Of course, to keep with the analogy we here assume counterfactually that *homo erectus* might still be alive today. Applied to *knowledge*: we should not think of *protoknowledge* as the core or essence of *knowledge* just because we have a predictively successful model that represents *knowledge* as developing out of *protoknowledge*.^3^

Having summarized our interpretation of Craig’s approach, we now turn to some important objections in the literature. In each case, we will offer strategies for defending Craig. In so doing, we will invariably go beyond his own words. And we are unsure whether he would approve of our arguments.^4^

## Functions

### Objection 1

Craig’s central hypothesis is that the core function of knowledge attributions is to flag good informants. Other authors have drawn attention to different functions:

- Signalling that inquiry is at an end (Kappel 2010; Kelp 2011; Rysiew 2012).
- Identifying propositions we can treat as reasons for acting (McGrath 2015).
- Providing assurance (Austin 1946; Lawlor 2013).
- Distinguishing between blameless and blameworthy behaviour (Beebe 2012).
- Honouring the subject of knowledge attributions (Kusch 2009).

Some of these authors intend their proposals to be alternative claims about the *central* role of knowledge attributions (Kappel, Kelp, Rysiew, Lawlor), while others intend them to be complementary (Beebe, Kusch, McGrath).

*Reply: *There is more than one way for a ‘Craigean’ to answer this objection.

First, although Craig does not explicitly discuss alternative genealogies, there is no reason to interpret him as holding that all knowledge attributions serve a *single *function. Here it is helpful to remember that Craig’s first publication on the genealogy of the concept *knowledge* was entitled ‘The Practical Explication of Knowledge’ (1986). ‘Explication’ refers to Rudolf Carnap’s work. Carnap allows for more than one explication of a given concept (e.g. 1950: pp. 1–18), but he also suggests some dimensions along which different explications might be evaluated: “(1) similarity to the explicandum, (2) exactness, (3) fruitfulness and (4) simplicity” (*ibid.*: 5). It would be an interesting exercise to compare the different hypotheses concerning the core functions of knowledge attributions on this basis. Alas, it is difficult to do so as things stand. The problem is that Craig has offered, in two book-length volumes, a detailed and extensive argument in favour of the fruitfulness and simplicity of his proposal. That is, he has tried to establish that his proposal meets the conditions of internal and external validity: internal, insofar as the evolution within the model is plausible; external, insofar as the proposal predicts the intuitions and their elaboration in a wide range of epistemological theories. None of the other proposals has been developed anywhere close to an equal level. Our claim is not that this would be impossible. Our claim is rather that this has not yet been done. The jury is out, and Craig’s proposal is innocent.

Second, an artefact can have two kinds of functions: *designated* (*standard, conventional*) or *accidental*. Take a screwdriver. Its designated function is to help us turn screws and bolts in such a way that they penetrate various hard materials. Screwdrivers can also serve other, accidental functions: e.g. as makeshift hammers, as murder weapons, or as muddlers. These functions are not widely recognized as constitutive of being a screwdriver. While one is usually aware which object or event serves which designated function in one’s own culture, the designated function is not always obvious (e.g. because the act of designation happened implicitly or in the distant past).

One important difference between designated and accidental functions is that whatever explains why some item can perform its designated function also explains the possibility of it performing additional, accidental functions. Screwdrivers need to be a certain shape to serve their designated function, and the shape of screwdrivers explains why we can use them as muddlers, not the other way around. Note that an artefact can have more than one designated function. A Swiss Army knife can function as a knife, a spoon, a saw or a pair of scissors. A beer glass can have the designated function of holding a liquid and of being beautiful to look at. In the former case, there is no ‘design hierarchy’ between the different functions: all designated functions of the Swiss Army knife are ‘equal’. In the case of the beer glass the designated function of holding a liquid is prior to the looking beautiful; it constrains and sometimes explains the secondary function.

‘Flagging good informants’ is, on Craig’s account, a designated but non-obvious function of our concept of *(proto)knowledge*. The designation in this case was not an identifiable explicit act in the past, and is not obvious to the casual observer—philosopher or layperson. This is why it needs Craig’s extensive discussion. Of course, this does not preclude that *(proto)knowledge* might also have other functions. Some of these other functions might be designated, some accidental. On the one hand, it is an accidental function of knowledge attributions that they can be used as words of solace (‘I know what you are going through’). On the other hand, ‘signalling that inquiry is at an end’ is too frequent a function of knowledge attributions for Craig to count it as accidental. It is probably best to count it as designated. But this raises the question whether ‘signalling that inquiry is at an end’ and ‘flagging good informants’ relate to one another as spoon and saw of a Swiss Army knife, or as holding liquid and looking good in the case of the beer glass. Opting for the former is to allow that *(proto)knowledge* has at least two distinct designated functions, neither of which is reducible to the other. Again, there is no reason why the genealogy of *knowledge *should rule out this option. One can see the different designated functions as complementary.

Third, one might argue that either ‘signalling that inquiry is at an end’ or ‘flagging good informants’ is the more basic designated function and thus explains the presence of the other derived designated function. The genealogist might argue that ‘termination of inquiry’ function flows naturally from the good-informant function: after all, the good informant is precisely the person who allows the inquirer to terminate her inquiry (Kusch 2013). His opponent argues in the opposite direction: one important way of signalling that inquiry can be terminated is to identify someone who has the information that can terminate it (Rysiew 2012).

Is there anything that can break this impasse? The genealogist might suggest that flagging good informants seems more important than indicating when one can stop inquiry. While we do need to stop inquiring at some point, there is no obvious need for a general term for the whole—potentially highly context-sensitive—class of such stoppings. In contrast, acts of flagging informants have an audience. We cannot flag someone without a flag! Will that convince the detractors? Maybe not. Williams (2015) argues that we need ‘knowledge’ as an explicit marker only once we have reached the phenomenon of ‘mature human knowledge’; this is a form of knowledge essentially tied to, and constituted by, an epistemic institution for marking ‘accountability, due diligence and liability to sanction’. As Williams’ sees it, in Craig’s state-of-nature the inquirers need not be reflective and explicit about their needs. Their needs for information could be served without an explicit epistemic vocabulary (Williams 2015: p. 257).

To sum up our answers to Objection 1: As things stand, Craig’s genealogy is the most elaborate theory about the designated function of knowledge attributions. Other, alternative proposals have not yet been pushed to a point where one could compare their relative strengths and weaknesses as far as fruitfulness or simplicity is concerned. This does not mean, however, that Craig has given us reason to think that such alternatives could not be constructed. And there remains the important and plausible option that knowledge attributions have more than one *irreducible designated* function.

## Paradigm cases

### Objection 2

There are paradigm cases of knowledge attribution where the function of flagging good information plays no role. For instance Mikkel Gerken says: “I might discuss Galileo Galilei’s intellectual development with my (also nerdy) friend who might assert ‘In 1616, Galileo knew that the Earth orbits around the sun”’ (2015: p. 226). But, he continues, this “knowledge ascription does not serve to assure me that the Earth orbits around the sun or that we can rely on Galileo with regard to this question” (*ibid*.) Or consider the following context. Samson tells his wife Sally that there is coffee in the pot. Samson thereby means to offer the coffee to Sally. However, Sally has told Samson many times that she hates coffee. In the context, she might convey a reminder with the words: ‘I know that there is coffee in the pot’. Gerken deems these “familiar uses of knowledge attributions in familiar conversational contexts” (*ibid.* 227). While Gerken uses these examples against Lawlor’s (2013) assurance-conception of knowledge attributions, what he says equally applies to Craig.

*Reply: *The distinction between paradigmatic and borderline cases of knowledge is tricky. For instance, Jonathan Kvanvig sees non-factive uses of knowledge attributions or expressions like ‘the current state of scientific knowledge’ as marginal uses of ‘knowledge’: the first is simple ‘misspeaking’ and the second is ‘merely honorific’. Such uses belong to the pragmatics, not the semantics of knowledge attributions (Kvanvig 2003: xi, pp. 190, 201). Other epistemologists disagree (Hazlett 2010; Kusch 2009, 2011). The argumentative standoff between such writers suggests that we lack clear criteria for deciding between paradigms and borderlines.

The immediate target of Gerken’s example is a kind of ‘function-first’ epistemology that tries to draw conclusions about the semantics of knowledge attributions directly from the claim that knowledge attributions serve a particular designated function (Hannon 2013; Henderson 2009; McKenna 2013). While these authors recognise that there may be other designated functions, the more uses of knowledge attributions that don’t serve the relevant designated function, the less plausible this argument looks. Let us consider the above examples in the context of such forms of function-first thinking. The claim about Galileo is a past-tense knowledge attribution. Sometimes a past-tense knowledge attribution is used to flag a good informant; for instance: ‘Trust me, so far I have always known where best to look for tasty berries.’ But, other times, it isn’t; in the Galileo case, the hearer already has the relevant information. Gerken is surely right about this.

If the genealogist is committed to insisting that knowledge attributions always and everywhere have the function of flagging good informants for the present audience, then Gerken has advanced decisive counterexamples. So Gerken’s examples show that even the function-first epistemologist à la Hannon, Henderson and McKenna had better allow for the possibility—after some objectivization of *knowledge* has occurred—that sometimes we speak about flaggings of good informants in the past, future or in counterfactual scenarios. In making such claims, we consider whether someone was, will be, or might be, a good informant for present, past, future or hypothetical audiences. Taken in this way, the claim about Galileo would amount to something like: ‘In 1616, Galileo was a good informant about whether the Earth orbits the Sun.’ Sally’s case must be handled differently. Couched in the good-informant format, Sally is saying to Samson that she is a good informant about whether there is coffee in the pot. The pragmatic context suggests what Samson is to infer from this: Sally is in no need of a good informant about whether there is coffee in the pot; and that this is because Sally does not like coffee.

At any rate, as our replies to Objection 1 suggest, we think the genealogist has good reasons for accepting that knowledge attributions serve *more than one designated* function, and that (some of) these functions are irreducible. This does not open the door to an ‘anything goes’ genealogy. After all, genealogists follow Carnap and evaluate different proposals for designated functions according to their fruitfulness (and other virtues). Craig is best read as insisting that if we take flagging good informants to be the designated function of knowledge attributions—the reason why we have the institution of knowledge attributions in the first place—then we can give an account of how the concept of *knowledge* developed that helps us to explain a wide range of the intuitions underlying the various theories of knowledge. This project succeeds if Craig can indeed explain these intuitions. This reveals an interesting aspect of Craig’s methodology: he uses a hypothesis about the function served by a subset of knowledge attributions to explain why we endorse accounts of knowledge that then can be applied in cases where the relevant function is not performed.

## Naturalized epistemology and the genealogy of ***knowledge***

### Objection 3

Hilary Kornblith interprets Craig as maintaining that knowledge is not a *natural kind* like water or aluminium, but an *artificial kind* like table or monarchy (2011: pp. 43–44). Kornblith bases this reading on one central passage: “Knowledge is not a given phenomenon, but something that we delineate by operating with a concept which we create in answer to certain needs, or in pursuit of certain ideals” (Craig 1990: p. 3). Kornblith disagrees: knowledge is a natural kind since the category of knowledge plays a significant explanatory and predictive role in one particular natural science: *cognitive ethology* (the science of animal behaviour). Moreover, just as chemists rightly ignore folk concepts of *water* or *aluminium*, epistemologists should pay little attention to folk concepts of *knowledge* or *justification* (2006: p. 12). Kornblith adopts Richard Boyd’s conception of natural kinds, on which they are homeostatic property clusters (Kornblith 2002: p. 61; cf. Boyd 1999). Applied to knowledge this suggests the following formulation:

The knowledge that members of a species embody is the locus of a homeostatic cluster of properties: true beliefs that are reliably produced, that are instrumental in the production of behavior successful in meeting biological needs and thereby implicated in the Darwinian explanation of the selective retention of traits (Kornblith 2002: p. 62).

Kornblith does not deny that knowledge (or the concept *knowledge*, or the institution of knowledge attribution) has social roles. But this is compatible with its being a natural kind. Natural kinds can have social roles. Gold is a case in point. Moreover, the social role of gold can, at least in good part, be explained by its natural properties (e.g. the relative rarity of gold) (2011: p. 45). And he does not regard the flagging of good informants as essential to knowledge. Animals can know things, but they do not flag good informants (2011: p. 46).

*Reply: *Kornblith makes two, closely related, criticisms. The first is that knowledge is a natural kind, so Craig is wrong in treating knowledge as an artificial kind. Unfortunately, this criticism is based on a problematic use of Boyd’s conception of natural kinds. Kornblith writes as if there were only *one single natural kind of knowledge*: knowledge is what cognitive ethology tells us it is. But Boyd denies that there is *one unique set* of natural kinds (Boyd 1999: p. 160). Natural kinds are relative to “disciplinary matrices” (scientific disciplines or groups thereof). These disciplinary matrices are constituted in part by human interests, projects and practices and thus these interests, projects and practices are “partly definitive of natural kinds” (Boyd 1980: 642–643). Moreover, Boyd thinks that intellectual history and the social sciences have natural kinds too. Thus feudal economy, capitalism, Islam, or Empiricism, are all natural kinds (Boyd 1999: pp. 154–156, 162–164). Kornblith can therefore be criticised from a Boydian perspective: Why isn’t the kind knowledge used in the *sociology of knowledge* also a natural kind? For the sociologist of knowledge, knowledge is a “purely ... a natural phenomenon.” Knowledge is “whatever people take to be knowledge.” Knowledge consists of “those beliefs which people confidently hold and live by ... which are taken for granted as institutionalised, or invested with authority by groups of people.” Knowledge is “what is collectively endorsed” (Bloor 1991: p. 5). This natural kind of knowledge does not coincide with the natural kind of knowledge investigated by cognitive ethologists. But this does not weaken its credentials within the disciplinary matrix of the social sciences.^5^

Kornblith’s second criticism is that, because knowledge is a natural not an artificial kind, Craig puts far too much weight on our intuitions about knowledge. But this criticism is only plausible if knowledge is a natural (-scientific) kind as opposed to a natural (social-scientific) kind. As Kornblith points out, chemists do not study water by studying our concepts of water (2007: p. 39). (It is however worth noting that concepts are—at least by Boyd’s reckoning—natural kinds too: they are central in the disciplinary matrices of cognitive psychology, linguistics, and the philosophy of mind). But social scientists do study social kinds such as ‘democracy’, ‘Islam’ and ‘empiricism’ by considering how the historical ‘actors’ understood these categories. Boyd’s rejection of intuitions about natural kinds is defensible; his rejection of intuitions about social kinds is, at best, highly contentious.

Our objections to Kornblith’s own proposal lessen the weight of his criticisms of Craig’s genealogy. But we can also contrast the two projects in a more direct way. For Kornblith—as for Boyd—the central function of the concept of *knowledge* is to play a role in explanation and prediction. However, at least with regard to the first stage of his model—the static model of the epistemic state of nature—Craig rejects the focus on the need to explain others’ behaviour, and instead picks as central the need to flag good informants:

... the wish to explain, in some fashion, the behaviour of one’s fellows, ... (It ... has been suggested to me, that this idea could help us to see the concept of knowledge as some sort of theoretical construct, useful for explaining why other members of our community behave as they do.) But just how widespread this concern with explanation is ... is very hard to say ... it would not be advisable to allow ourselves such a starting point before we have exhausted the potential for far less contentious claims about the human situation ... (1990: p. 5).

This emphasis does mark a difference with Kornblith—but only as far as the first of Craig’s two models is concerned. Craig’s *dynamic* model (or at least a further development of it) leads to the prediction that further needs—in addition to the need to flag good informants—will also leave their marks on the concept. Taking Smith to be a good informant regarding the location of tigers enables you to explain and predict some of his actions. There thus is a natural route from *knowledge* as a flag for a good informant to *knowledge* as essential to explanations of actions.

And yet, is there not a deep divide between Craig and Kornblith insofar as Craig speaks of knowledge as “... something that we delineate by operating with a concept which we create in answer to certain needs ...”? Does this not, as Kornblith alleges, really exclude the option that knowledge might be a natural kind? No, it does not. As Boyd emphasises: “... in a certain sense, human interests, projects and practices are partly definitive of natural kinds” (1980: p. 642). We build disciplinary matrices to satisfy certain of our needs, and the explanatory, predictive and practical aims of disciplinary matrices determine which are the relevant concepts of natural kinds. This gives Craig all he needs to maintain his claim.

## Knowledge-first, function-first

### Objection 4

In his influential study *Knowledge and Its Limits*, Timothy Williamson argues that the traditional project of reductively analysing knowledge in terms of other notions (belief, truth, justification, Gettier-conditions) has failed. Call this his ‘negative claim’. Williamson furthermore proposes that knowledge is primitive, and the most basic factive mental state. Other epistemic categories can be illuminated (though not reductively analysed) by taking knowledge as the starting point. For instance, (mere) belief is something like “botched knowledge” (Williamson 2000: p. 47). Call this the ‘positive point’. In a footnote Williamson acknowledges that Craig agrees with him on the negative claim, but laments Craig’s failure to go along with the positive point. Williamson suggests that accepting the positive point would undermine Craig’s whole project:

Craig 1990 makes an interesting attempt to explain the point of the concept of knowledge in the light of the failure of analyses of the standard kind. However, on the present view it remains too close to the traditional programme, for it takes as its starting point our need for true beliefs about our environment (...), as though this were somehow more basic than our need for knowledge of our environment. It is no reply that believing truly is as useful as knowing, for it is agreed that the starting point should be more specific than ‘useful mental state’; why should it be specific in the manner of ‘believing truly’ rather than in that of ‘knowing’? (2000: p. 31).

*Reply: *We suspect that this dispute between Williamson and Craig is going to be hard to resolve. One immediate response would be: why think that our need for true beliefs about our environment is *not* more basic than our need for knowledge? Williamson’s objection seems to straightforwardly beg the question against Craig. But this response doesn’t take us very far because the idea that knowledge is (in some sense) more basic than true belief is at the very heart of Williamson’s ‘knowledge-first programme’. To the extent one goes along with this programme, the objection has real force (and, to the extent one doesn’t, it has little force). We are not going to be able to settle the case for or against the knowledge-first programme here. Instead, we will make some comments about how we see the relationship between Craigean genealogy and knowledge-first epistemology.

First, it is important to recognise that Craigean genealogy removes one of the motivations for knowledge-first epistemology. Craig agrees with Williamson about the (negligible) prospects for the traditional project of giving a reductive analysis of knowledge in terms of other notions. In doing so he undercuts one of the central arguments for the knowledge first programme in Chapter 1 of *Knowledge and its Limits *(Williamson 2000): why not take the failure of the traditional project to show that knowledge is primitive? Craig offers us an alternative: why not take the failure of the traditional project to motivate giving a genealogy of knowledge?

Second, while Williamson doesn’t put it this way, it is tempting to read him as proposing that knowing qua factive mental state is a natural kind. Assume it is a natural kind of the philosophy of mind.^6^ What sort of conception of natural kinds might the Williamsonian appeal to? One option would be to the Boydian account of natural kinds discussed in the previous section. But then we can just apply what we said about Kornblith here too. Why should we believe that this is the *only* scientifically or philosophically legitimate conception of knowledge? Are there not also other scientific disciplinary matrices that achieve their explanatory-predictive goals by postulating other conceptions of knowledge as their natural kinds?

Another option would be the Kripke/Putnam account of natural kinds (see Kripke 1980; Putnam 1975). So let us compare Williamson’s conception of knowledge as a natural kind with a natural kind like gold. Gold has two kinds of properties: essence-defining properties concerning its microstructure (atomic number 79), and superficial properties such as that it is shiny, rare, occurs in rocks or as nuggets, etc. Kripke and Putnam famously argue that the relationship between these two sets of properties is as follows: the superficial properties were used to fix the reference of the term ‘gold’, and then science discovered the essence of gold. But how well does the Kripke–Putnam model fit with Williamson’s conception of knowledge? We would need to hold that the superficial properties of knowledge (whatever they are) fixed the reference of the term ‘knowledge’, and then epistemology (in particular, knowledge first epistemology) discovered the essence of knowledge: knowledge is the most basic factive intentional attitude. Now, this would be a genuine discovery, perhaps on a par with the discovery of the chemical structure of gold. But why think that Williamson’s investigations into knowledge discovered its essential nature, or more generally, that being the most basic factive intentional attitude is part of the essential nature of knowledge? It’s not as if ‘hard to discover’ properties are generally essential properties.

Third, let’s assume that there is a response to all these questions and that Williamson can plausibly maintain that knowledge is a natural kind. Does this show that Craig’s genealogical investigations are pointless? It is hard to see why. It is surely an interesting question to ask how people came to pick out certain objects or materials in their environment; what functions gold and gold-talk had in their communities; and how different groups of specialists used gold (and gold-talk) for different purposes. Sometimes these investigations might even challenge the Kripke–Putnam model: It may turn out that intuitions and linguistic practices do not always follow the lead of science. Some communities might for instance decide to let the term follow the appearance—the surface properties—rather than the microstructure. Applied to knowledge: even if knowledge is a natural (psychological) kind we can ask Craigean questions about how people came to pick this natural kind out; what functions knowledge and ‘knowledge’-talk had and have in our communities; and how different groups of specialists (e.g. epistemologists) used knowledge (and ‘knowledge’-talk) for different purposes. If we are right about this, then the Craigean and the Williamsonian need not be enemies; they can be friends. We do not need to decide which project comes first; they are both important, albeit for different purposes.

Still, one might worry, as one of our reviewers put it, that this ‘friendship’ will leave the Craigean with only a ‘minimal project’. Take water. Presumably *water* and ‘water’ have genealogical histories; it is just that these histories tell us nothing important about the nature of water. And mutatis mutandis for knowledge.’

We beg to differ. The genealogical history of ‘water’ or *water* is far from trivial, and of considerable philosophical significance. Take for instance Jamie Linton’s recent book *What is Water? The History of a Modern Abstraction* (Linton 2010). Linton traces the reduction of*water* to $H_{2}O$ and describes this process as one that has “essentialized water to the point where it is extracted from the social contexts of human experience and treated as an invariant essence” and as a mere “resource” (2010: p. 41). In other words, Linton seeks to offer a way of “analysing both the history of water and how the idea of water articulates with its material and representative forms to produce this history” (ibid.). Or consider Hasok Chang’s study *Is Water*$H_{2}O?$ (2012): Chang tells the story of how we came to identify water with $\hbox {H}_{2}\hbox {O}$, how this identification was contingent upon specific scientific controversies and their resolution. Chang in fact argues that there was a loss in the understanding of water when it was equated with $\hbox {H}_{2}\hbox {O}$. We do not need to take a stand on the details of Linton’s or Chang’s genealogies. But surely it is of philosophical interest to learn about the importance of phenomena not included in the essence when these phenomena have impacts on how we live.

It should be clear how we wish to transfer these ideas to the Craigean project: it is far from obvious that the genealogy of *knowledge* would be a minimal project of little philosophical interest if knowledge were a natural kind. Is it so clear, for instance, that phenomena of epistemic injustice are best analysed against the backdrop of a Williamsonian primitive natural kind view rather than against the backdrop of a Craigean genealogy? Miranda Fricker’s influential study of epistemic injustice (Fricker 2007) takes place precisely against the backdrop of a Craigean genealogy. It seems to us that Fricker’s investigation would lose nothing of substance if she accepted a Williamsonian picture on which knowledge is a psychological kind.

## Constraints on genealogies

### Objection 5

Craig gives us a *fictional* genealogical account of the development of the concept of *knowledge*. Central in this account is his *hypothesis* that the concept of *knowledge* served a certain function. He assumes that knowledge attributions served this function in a *fictional* state of nature. But what can such fictions tell us about reality? And what are the constraints on genealogies? Can we make them up as we make up works of fiction in general? Or as Miranda Fricker puts it: “It is at the best of times difficult to grasp the status of state-of-nature stories. They are notorious for providing a blank canvas onto which a philosopher may paint the image of his personal theoretical predilections” (1998: p. 164).

*Reply: *Our answer to Objection 5 is implicit in our outline of the overall project and our replies to earlier objections. Craig is engaged in the kind of modelling familiar from the natural and social sciences. Models invariably idealize. Philosophers of science standardly distinguish between two forms of idealization: in ‘Aristotelian idealization’ inessential features of a ‘target system’ (the physical system under study) are stripped away. Only those features that are relevant to the occurrence and behaviour of the phenomena of interest are included in the representation (Cartwright 1989). In ‘Galilean idealization’ some features of the target system are deliberately distorted. The representation operates on the basis of assumptions about that system which are known to be false (McMullin 1985). ‘Caricatures’ combine Aristotelian and Galilean idealizations (Frigg and Hartmann 2012). Finally, for our purposes it is useful to add a third form of idealization that might be called ‘Wittgensteinian’. This form of idealization is in play when one restricts one’s investigation to a very small (potentially ‘one-sided’) ‘diet’ of examples (cf. Wittgenstein 1953: p. §593). Models are measured by their internal and external validity: is the process claimed to unfold in the model plausible and tractable, and does the model make correct predictions concerning the target system?

Clearly, Craig’s genealogy idealizes in all these respects. As we read him, Craig describes a process of gradual objectivization that starts with a relatively simple concept (*protoknowledge*) and leads to a more complicated family resemblance concept (*knowledge*). One can ask various questions about this process. Cognitive psychologists or experimental philosophers might want to test whether the development of children’s understanding of knowledge is as the Craigean hypothesis of objectivization predicts. Maybe anthropological data also bears on the question. The prior probability of this hypothesis seems high enough to merit attention. But its truth is not crucial for Craig’s method. Remember that we are concerned with *models*, and models inevitably idealize and possibly even distort. The key question is: are Craig’s models internally and externally valid? To be externally valid they must make sense of the intuitions underlying different theories of knowledge, and ‘deliver’ a concept of *knowledge* that is extensionally and intensionally equivalent (or at least close) to our concept of *knowledge*. This is not the place to assess how well Craig’s models do in this respect. Our point is merely that the external validity of the models can be rationally assessed. As far as internal validity is concerned, the conceptual synthesis should be plausible both philosophically and when judged by the results of, say, historical linguistics. Moreover, remember that Craig ties the development from *protoknowledge* to objectivized *knowledge* to specific social patterns. For instance, a more objectivized conception of *protoknowledge* becomes important as people have to flag good informants to a wider circle of other inquirers. This shows that the internal validity can also be evaluated in light of social theory or social ontology.

Fricker and Bernard Williams challenge Craig precisely on the final point. Williams (2002) questions Craig’s assumption that the inhabitants of the epistemic state of nature are cooperative. As Williams sees it, cooperation cannot be taken for granted in this way. Hence Williams insists on, and develops, an account of how information-sharing is possible as a social institution and collective good. Fricker finds both Craig’s and Williams’ characterizations of the epistemic state of nature insufficiently *political*: neither of them says anything about the effects of social categorization upon one’s status as a good or bad informant (Fricker 2007). We agree with both criticisms. But we also agree with these two critics that these problems can be overcome through a further development of Craig’s general approach.

## Contextualism and relativism

### Objection 6

Several authors have argued that Craig’s genealogy supports a contextualist semantics for knowledge attributions (Greco 2007; Hannon 2013; Henderson 2009; McKenna 2013). More recently, John MacFarlane (2014: pp. 311–319) has argued for a close connection between genealogy and relativism about knowledge attributions. They can’t both be right.

*Reply: *It is easy to see why Craig’s genealogy might seem to support contextualism. Put roughly, contextualists about knowledge attributions hold that the word ‘know(s)’ is context-sensitive, in much the same way as indexicals like ‘I’. Just as what it means to say ‘I am tired’ depends on who is speaking, what it means to say ‘S knows that p’ depends on the ‘epistemic standards’ of the speaker. Thus, in contexts where ‘high’ epistemic standards are appropriate (e.g courtrooms), to say someone ‘knows’ is to say that they know by high standards; in contexts where ‘low’ epistemic standards are appropriate (e.g. bars), to say someone ‘knows’ is to say that they know by low standards. This fits with some aspects of Craig’s genealogy because it seems clear that whether a subject is a good informant depends on and varies with the context. If I’m talking to a friend over lunch about Isla’s whereabouts last night and I have good but not conclusive evidence that she was at the party I’ll volunteer myself as an informant on her whereabouts. However, if I’m giving a statement to the police and I have the same evidence I’ll not volunteer myself as an informant on her whereabouts. Consequently, given that what one will require of a good informant depends on and varies with the context, what it means to say someone ‘knows’ must depend on and vary with the context too.

There are however reasons to think that things are not so simple. Contextualism poses problems for the transmission of knowledge through testimony, and so doesn’t fit with the obvious importance of testimony to Craig’s account. The problem is that, if uses of ‘know(s)’ mean different things in different contexts, then knowledge attributions cannot perform this valuable public service (Hawthorne 2004). I may be told that Isla said that Morven knows the bank is open, but unless I also know what epistemic standards Isla was using, this information is of little use. Compare: I may be told that somebody said ‘I am tired’ but, unless I know who said this, this is no help if I want to keep track of who is tired. In general, our ability to make use of reported claims using indexicals requires us to be able to assign those claims definite contents.

For related reasons MacFarlane thinks that Craig’s genealogy supports relativism. As MacFarlane construes the issue, the difference between contextualism and relativism is easiest to grasp by focusing on past knowledge attributions and their ‘contexts of use’, and how these might be assessed in later contexts (i.e. ‘contexts of assessment’). The contextualist insists that the latter assessment must always keep to the standards that were operative in the original context of use (of the respective knowledge attribution). The relativist instead holds that the only standards that count are the later standards. MacFarlane thinks that the relativist semantics is the more plausible since it makes fewer demands on our memory: “There would be no need to store a standard with each knowledge attribution, because all of the knowledge attributions would be evaluated in relation to the current standard” (2014: p. 312). To hammer home the point MacFarlane even offers his own ‘evolution of assessment sensitivity’: it may well have been that “once upon a time, ‘knows’ behaved just as contextualists say it does” (317). But as social interaction increased and knowledge attributions were exchanged ever more widely across situation and standards, it simply became too tedious to keep track of the standards attached to each attribution. And thus speakers drifted towards the relativist understanding.

We have both a case for tying genealogy to contextualism, and a case for tying it to relativism. Which case is stronger? This question is of course legitimate. But there is also a sense in which it is not fully in keeping with Craig’s project. Craig’s goals are primarily *descriptive* (trying to explain the intuitions we in fact have) rather than *normative *(trying to build a theory on the basis of our intuitions). When it comes to the big oppositions in epistemology—internalism versus externalism, scepticism versus anti-scepticism—Craig’s primary goal is less to pick winners (though he does have his favourites), but to explain why both kinds of accounts have naturally arisen given the models of the good informant. And it seems possible to use genealogy for that purpose here. MacFarlane has given us a plausible (quasi-genealogical) account as to why we should expect to have intuitions supporting relativism. But there is also a plausible story to be told that goes the other way. MacFarlane makes much of the memory overload resulting from keeping track of the standards operative in the earlier context of use. The contextualist might respond by suggesting that these demands have compensating benefits. Keeping track of the epistemic standards individuals were using when they made knowledge attributions helps us figure out what to do with their knowledge attributions: ‘X said that S knows that p, but X was using low standards, so I can’t use S as an informant in my high standards situation’. Reasons for accepting a contextualist semantics for knowledge attributions are also reasons for thinking that any burdens that semantics places on speakers are worthwhile.

## Relativism and contingency

### Objection 7

The issue of relativism arises in a different way. Craig, in effect, relativizes our concept of *knowledge* to contingent aspects of human psychology and the structure of human societies. If Craig is right, there is nothing *inevitable *about the fact that we have the concept of *knowledge* we have. While Craig’s starting point is a supposedly universal human need—for picking out and flagging good informants—this need is universal only within the human species. Omniscient creatures would have no need for a way of identifying good informants; they would have all the information they need at their fingertips. Further, even given a common universal starting point for the human species, Craig’s genealogy does not guarantee that all epistemic communities give the same weight to each and every step of the (possibly co-existing) stages of development from *protoknowledge* to *knowledge.* And, to make matters worse, there are also those genealogists who argue that Craig’s ‘imaginary genealogy’ needs to be complemented by ‘real genealogy’, that is, by an engagement with historical and cultural contingent realities. Bernard Williams insists that in moving from imaginary to real genealogy we do not leave philosophy behind: “... philosophy cannot be too pure if it really wants to do what it sets out to do” (2002: p. 39). Whether it is philosophy or not, the worry is that relativity will multiply as these historical and cultural contingencies enter the picture.

*Reply: *It is unclear whether this form of relativism should worry the epistemic absolutist. Once again, it is important to remember that Craig’s project is (primarily) descriptive, not normative. Relativism seems most worrying when it tells us that we *should* think of all different systems of epistemic standards as somehow ‘equally valid’ (maybe in the way in which we might think of different tasty- or funny-judgements as being equally valid). But genealogy does not tell us what we *should* do. It ‘merely’ explains why we have the intuitions that we do have. So Craigean genealogy does not entail any claims to the effect that different systems of epistemic standards are in any sense ‘equally valid’. It thus does not fall prey to the objections levelled against relativism in books like Paul Boghossian’s *Fear of Knowledge* (2006).

And yet, in our view genealogy does have a normative dimension. It does not ‘leave epistemology as it is’. This brings us to an issue we have suppressed up to this point: Craig’s own, rather late, reminder that genealogies can be ‘subversive’ or ‘vindicatory’ (2007: p. 182). The former undermine a view by revealing its disreputable origins (Nietzsche), the later support it by displaying its good or acceptable beginnings (Hobbes). Craig makes little of this distinction in his work from the 1990s, but we wonder whether it might not potentially be significant in the current context. Think of it this way: the absolutist about epistemic norms must make plausible the idea that our existing epistemic standards (for attributing knowledge) track the one absolutely correct epistemic system of norms (e.g. Goldman 2010). A Craigean genealogy might be taken to debunk this sort of picture of epistemic norms. Does genealogy not undermine the idea of ‘context-free or super-cultural norms’ (Barnes and Bloor 1982: p. 27)? Does it not subvert such notions? We are inclined towards a positive answer, but the question clearly demands further investigation.

## Conclusion

Craig’s genealogical method has spurred a lot of epistemological discussion. But there has been surprisingly little attention paid to some of the objections that have been raised against Craig’s method. In this paper we have considered seven objections from the literature and outlined how someone attracted to the genealogical method might respond to them. Most of these objections don’t purport to be ‘knock-down’ objections; similarly, most of our responses don’t purport to end discussion of these issues. In our view, the variety of functions of knowledge attributions (Objection 1), the differences with ‘knowledge first’ epistemology (Objection 4) and the issue of relativism (Objections 6 and 7) merit particular attention. But we hope this paper has shown that Craig’s method remains viable, and has a lot to offer contemporary epistemology.

## Footnotes

1 A note on terminological conventions. We use italics for concepts (*knowledge*, *protoknowledge*) and quotation marks for words (‘knowledge’). When we are talking about the thing itself, or it is unclear whether the author we are discussing means to refer to the concept, word or the thing itself, we just write <knowledge>.

2
Beebe (2012), Dogramaci (2012), Fricker (1998, 2007), Gardiner (2015), Gelfert (2011), Gerken (2015), Greco (2007), Hannon (2013), Henderson (2009), Kappel (2010), Kelp (2011), Kornblith (2011), Kusch (2009, 2011, 2013), Lawlor (2013), MacFarlane (2014), McGrath (2015), McKenna (2013), Neta (2006), Pritchard (2012), Reynolds (2012), Rysiew (2012), Williams (2002, 2015).

3 Does this imply that in everyday life we use the concept *protoknowledge* to identify good informants? If so, one might object that this seems highly unlikely—no ordinary speakers have the concept *protoknowledge*. Instead, they use the concept *knowledge*. But this misses our central point. To switch metaphors, Craig’s account suggests that *knowledge *is a family resemblance concept, and that in different situations different family members become relevant. Thus sometimes we operate with something like *protoknowledge*, but *protoknowledge* is one family member of the family resemblance concept knowledge. The other family members can be distinguished conceptually by using a model that postulates a development in which *protoknowledge* is increasingly objectified.

4 We set aside two objections because, in our view, Craig’s response to them can’t be improved on. The first is what Craig calls the ‘cart before the horse’ objection: namely, the fact that we can imagine knowers who are not good informants, or good informants who are not knowers, shows that we can’t make sense of someone being a knower in terms of them being a good informant. Craig’s response is that we can illuminate why we have a concept by holding that it was introduced to serve a need without holding that every application of the concept serves that need, or that only applications of the concept serve the need (see 1990: p. 95). The second is that there is a tension at the heart of Craig’s genealogy. The process of objectivization transforms the ‘subjective’ concept of someone who is likely enough to be right for *my *purposes (a *protoknower*) into the ‘objective’ concept of someone who is likely enough to be right for *anyone’s *purposes (a *knower*), and it is rare indeed for someone to be likely enough to be right for anyone’s purposes. But it is precisely because this tension exists that Craig thinks he can diagnose both why the sceptical problem arises and why it is so difficult to solve (see 1990: p. 119, 1993: pp. 116, 148).

5 One might object that, in the sociology of knowledge, ‘knowledge’ really designates a kind of belief (collectively endorsed belief), not the natural kind Kornblith is theorizing about. But for Kornblith knowledge is also a kind of belief: reliably produced true belief (see Kornblith 2002). The difference between Kornblith and the sociologists of knowledge concerns what we need to ‘add’ to belief to get knowledge, not whether knowledge is a kind of belief. While we agree that the sociology of knowledge is not talking about the natural kind Kornblith is theorizing about, we take this to motivate a sort of *pluralism*: there may be a plurality of natural kinds here, and there is no reason to focus on one of these natural kinds at the expense of the others (see Kusch 2013).

6 We can bolster this interpretation by looking at what Williamson says about the role of knowledge in psychological explanation. To use his example, that the burglar knew the diamonds were in the house may be a better explanation of why they spent all night ransacking the house than that they merely believed (or truly believed) that the diamonds were in the house because the probability of the ransacking occurring conditional on the burglar knowing is higher than of it occurring conditional on their merely believing (or merely believing truly) (see Williamson 2000: p. 62). One might take this to show that knowledge plays a crucial role in psychological explanation, and therefore is a sort of psychological kind.
